# Platelet-rich fibrin and titanium-prepared platelet-rich fibrin in endoperio lesion management

**DOI:** 10.6026/97320630019133

**Published:** 2023-01-31

**Authors:** Balram Choudhary, Komal Goswami, Bhumika J Patel, Ansee R Vaghani, Dhurubatha J, Nishita Grandhi, Ramanpal Singh Makkad

**Affiliations:** 1 Department of Dentistry, JLN Medical College and Hospital, Ajmer, Rajasthan, India; 2 Department of Dentistry, Kshetrapal Hospital, Ajmer, Rajasthan, India; 3 Department of Oral Medicine and Radiology, Gujarat, India; 4 BDS, Gujarat, India; 5 Department of Oral and Maxillofacial Surgery, Government Dental College, Kadapa, AP, India; 6 Department of Conservative and Endodontics, New Horizon Dental college and Research institute, Bilaspur, Chhattisgarh, India; 7 Department of Oral Medicine and Radiology, New Horizon Dental college and Research institute, Bilaspur, Chhattisgarh, India

**Keywords:** Endo-perio lesions, platelet-rich fibrin, titanium-prepared platelet-rich fibrin

## Abstract

Endo-perio lesions involve a variety of therapy choices that will lead to the best possible elimination of infection. Various therapy approaches have been investigated for curing of patients affected by endo-perio abnormalities. One of the second-generation platelet derivatives is plasma enriched with platelet (PRP).They may aid in the healing of wounds. Enhanced with platelets cells and several growth factors, platelet-rich fibrin (PRF) promotes repair and healing and regeneration of tissue at the periapical area. Platelet cell and leukocyte cell enriched fibrin, prepared in conjunction with titanium (T-PRF), is analogous to fibrin made using the traditional PRF process.The current study was undertaken to compare PRF against T-PRF in the therapy of endo-perio abnormalities using the basic information that was available.280 patients of all sexes between the ages of 18 years and 58 years make up the study's participants. They were divided into two categories. In category I study participants PRF was employed to fill the defect created due to pathology and in category II patients, a T-PRF was used, accompanied by suturing. The one walled, two walled, and three-wall walled infrabony abnormalities were quantified on the digital images acquired using the grid. After three months and six months, the probing periodontal pocket depth in mm and level of attachment (RAL) in mm were measured. In category one, mean change at 3 months was 3.21mm accounting for 33.79% change in PPD. On the other hand mean change at 6 months was 3.61mm accounting for 43.79% change in PPD. When there was evaluation in study participants in category two then it was observed that mean change at 3 months was 2.02mm accounting for 34.79% change in PPD. On the other hand mean change at 6 months was 3.62 mm accounting for 44.79% change in PPD. There was reduction of depth of periodontal pocket at both 3 months follow up and 6 months follow in both categories; however there was no statistical significant variation observed between the two categories regarding decrease in the depth of periodontal pocket on analysis of intergroup variations. It was concluded that there was increase in periodontal attachment and decrease in depth of periodontal pocket in both PRF and T-PRF however there was no statistical substantial variation observed between the two categories regarding increase in the attachment level or decrease in depth of periodontal when intergroup variations between PRF and T-PRF were considered.

## Background:

Periodontal ligament tissue as well as dental pulp tissue is both ectomesenchymal tissues that develop from dental follicle and dental papilla, respectively. Through apical foramen of root, lateral root canals and auxiliary canals, and tubules of dentin, there is direct interaction between the periodontal ligament tissue and dental pulp tissue. Periodontal pockets with greater probing depths, grooves that form during development and tubules of dentin can all correlate to the development of disease. [1] Therefore, the infection is transferred from the pulp tissue to the periodontium tissues. This infection is caused by a variety of bacteria. Mixed endo-perio lesions are challenging for professionals to treat because they are difficult to diagnose. Teeth with multiple roots typically have a bad outcome and are more vulnerable to infection. The periodontal tissue and root canal tissues both require treatment. [2] When there is no periodontal engagement, the overall performance of traditional endodontic therapy is substantially greater; however, in mixed endo-perio infections, particularly when regeneration steps are not taken, the overall performance rate is very low. Appropriate diagnosis is crucial for the management of patients. Better outcomes are guaranteed by the proper history of past illness, proper clinical examination and the radiographic analysis. [3] The co-existence of pulpal infection and periodontal infections might make the process of diagnosis and proper treatment planning difficult. In certain situations, the tooth pulp's pathological status may not be shown by the vitality test. On rare occasions, the diseased root canal's persistent inflammatory reaction might spread into the sulcus of gingiva and discharge through the sinus passages. In the absence of regional periodontal causes, the periodontal symptom often heals quickly following root canal therapy. Endo-perio abnormalities involve a variety of therapy choices that will lead to the best possible elimination of infection. [4] Various therapy approaches have been investigated for curing of patients affected by endo perio abnormalities. [5] Studies involving root excision procedure , retrograde filling procedure, and open flap cleaning procedure have been carried out by scientists. Modern tactics include regenerative techniques like application of tissue growth promoters, bone transplants, and guided tissue regeneration procedure (GTR). One of the second-generation platelet derivatives is plasma enriched with platelet (PRP). A considerable amount of platelets are present in the autologous fibrin matrix that makes up PRP. [6] They may aid in the healing of wounds. Enhanced with platelets cells and several growth factors, platelet-rich fibrin (PRF) promotes repair and healing and regeneration of tissue at the periapical area. Platelet cell and leukocyte cell enriched fibrin, prepared in conjunction with titanium (T-PRF), is analogous to fibrin made using the traditional PRF process. [7] Titanium has the greatest toughness proportions among metals and is resistant to corrosion. Because it is noncorrosive, it has excellent biocompatibility properties. Additionally, the titanium exhibits a remarkable osseointegration quality. [5] The current study was undertaken tocompare PRF against T-PRF in the therapy of endo-perio abnormalities using the basic information that was available.

## Material and Methods:

280 patients of all sexes between the ages of 18 years and 58 years make up the study's participants. We incorporated all individuals with endo-perio pathologies (main endodontic problem and secondary periodontal problem) in mandibular teeth with multiple roots, physiologically healthy individuals, and patients without a recent history of antibiotic use. Patients who were taking antibiotics, had recently undergone endodontic surgical procedure or periodontal surgical procedure, individuals who had habit of smoking tobacco, individuals who were pregnant woman or breast feeding mother, or patients who didn't give their agreement were excluded. Because molars and premolars of mandible are frequently affected by endo-perio pathologies, molars of mandible were chosen for the investigation. The study was explained to all registered patients, and their signed agreement was obtained in local dialect. To prevent bias in the findings, two experienced researchers conducted this double-blind research. For purposes of statistical analysis, the mean of findings of two researchers was used. There were two different categories of patients. Individuals who had the PRF administration were employed in category 1 and individuals who were administered with the T-PRF were assigned to category II. Two endodontists administered endodontic therapy to all of the study participants using uniform aseptic techniques. Before the periodontal phase of treatment, all affected teeth underwent endodontic therapy. The PRF was made by pipetting 10 mL of human blood from the human antecubital vein into a simple eppendorf tube, and then immediately centrifuging the sample in a desktop centrifugation device. The number of rpms was 3,000 and the duration of centrifugation was ten minutes. The procedure of centrifugation was carried out at room temperature. In order to prepare T-PRF, tubes of titanium were used to acquire fibrin-PRF. The number of rpms were adjusted at 2800.The duration of centrifugation was for twelve minutes. The flaw was carefully examined. In category I study participants PRF was employed to fill the defect created due to pathology and in category II patients, a T-PRF was used, accompanied by suturing. The Coe-Pack was set. All patients received postoperative recommendations. For fourteen days, a prescription was written for therapeutic drug Amoxicillin 500 mg, therapeutic drug Metronidazole 400 mg, therapeutics drug diclofenac sodium, and therapeutic drug 0.2% chlorhexidine mouthwash. The one walled, two walled, and three-wall walled infrabony abnormalities were quantified on the digital images acquired using the grid. After three months and six months, the probing periodontal pocket depth in mm and level of attachment (RAL) in mm were measured.

## Statistical Analysis:

The outcomes were then statistically evaluated using the unpaired t test and SPSS version 21.0 (IBM, Chicago, USA). The implication level was set as less than 0.05.

## Results:

Table 1: Probing pocket depth (mm) in both groupsIn this study PPD was measured in study participants at 3 months follow up and 6 months follow up. In category one mean change at 3 months was 3.21mm accounting for 33.79% change in PPD. On the other hand mean change at 6 months was 3.61mm accounting for 43.79% change in PPD. When there was evaluation in study participants in category two then it was observed that mean change at 3 months was 2.02mm accounting for 34.79% change in PPD. On the other hand mean change at 6 months was 3.62 mm accounting for 44.79% change in PPD. There was reduction of depth of periodontal pocket at both 3 months follow up and 6 months follow in both categories; however there was no statistical significant variation observed between the two categories regarding decrease in the depth of periodontal pocket on analysis of intergroup variations. (Table 1) In this study RAM (relative attachment level) was measured in study participants at 3 months follow up and 6 months follow up. In category one mean change at 3 months was 2.03mm accounting for 32.31% change in RAM. On the other hand mean change at 6 months was 3.63mm accounting for 41.79% change in RAM. When there was evaluation in study participants in category two then it was observed that mean change at 3 months was 2.92 mm accounting for 32.71% change in RAM. On the other hand mean change at 6 months was 3.52 mm accounting for 43.79% change in RAM. There was increase in attachment level periodontal at both 3 months follow up and 6 months follow in both categories; however there was no statistical significant variation observed between the two categories regarding increase in the attachment level on analysis of intergroup variations (Table 2).

## Discussion:

Traditional endodontic therapy performs significantly better overall when there is no periodontal involvement; but, in mixed endo-perio infections, especially when regeneration measures are not followed, the overall performance rate is quite poor. Correct diagnosis is essential for patient care. A correct medical history of prior illnesses, a correct clinical examination, and a precise radiographic study all ensure better results. [8] The diagnosis procedure and effective treatment planning may be challenging if pulpal infection and periodontal infection coexist. The vitality test may not always reveal the diseased state of the tooth pulp. Rarely,the root canal infection's ongoing inflammatory response may expand into the gingival sulcus and drain into the sinus passages. Plasma that has been enhanced with platelets is one of the second-generation platelet derivatives (PRP). The autologous fibrin matrix that makes up PRP contains a sizable number of platelets. [9,10] They might promote the recovery of wounds. Platelet-rich fibrin (PRF), which is enhanced with platelets cells and a number of growth factors, encourages tissue regeneration at the periapical area. When manufactured with titanium (T-PRF), platelet cell and leukocyte cell enriched fibrin is comparable to fibrin produced using the conventional PRF procedure [11, 12,13,14]. Among all metals, titanium has the highest toughness ratios and is corrosion-resistant. It has high biocompatibility qualities because it is noncorrosive. The titanium also has an amazing osseointegration quality. [15,16-17] Using the available fundamental data, the current study compared PRF and T-PRF in the treatment of endo-perio abnormalities. In this study PPD was measured in study participants at 3 months follow up and 6 months follow up. In category one mean change at 3 months was 3.21mm accounting for 33.79% change in PPD. On the other hand mean change at 6 months was 3.61mm accounting for 43.79% change in PPD. When there was evaluation in study participants in category two then it was observed that mean change at 3 months was 2.02mm accounting for 34.79% change in PPD. On the other hand mean change at 6 months was 3.62 mm accounting for 44.79% change in PPD. There was reduction of depth of periodontal pocket at both 3 months follow up and 6 months follow in both categories; however there was no statistical significant variation observed between the two categories regarding decrease in the depth of periodontal pocket on analysis of intergroup variations. 20 locations with three-wall faults were divided evenly into 10 sites for the investigation by Mitra et al. Patients in the group I used T-PRF, while those in the group II used PRF. At the beginning, three months later, and nine months later, clinical parameters such depth of periodontal pocket and level of clinical attachment were evaluated. Similar to our findings, the authors showed a significant decrease in depth of probing pocket and an increase in clinical attachment threshold in both categories from base point to 9 months. [17] When compared to PRF, T-PRF showed a denser fibril meshwork under scanning electron and light microscopy. Our findings concur with those of research by Chatterjee et al. and research by Mitra et al.[6,7,8, 9,10,11,12,13,14, 15,16,17] Three-walled intrabony periodontal osseous abnormalities were assessed utilising the technique of (OFD)open flap debridement, autologous PRF content, and OFD combined T-PRF by Chatterjee et al. Similar to our findings, they discovered no statistically relevant variation between the PRF category and T-PRF category in the outcome. 6 After applying the T-PRF membrane to rabbit specimens, Tunali et al. noted the development of new bone, new connective tissue, and significant wound healing over the course of 30 days. [5] A patient w as identified with endo-periolesion of the mandibular central incisor, according to Shashikumar et al. Scaling with root planning, or phase I therapy, was organised and was succeeded by traditional root canal therapy. After undergoing periodontal surgical procedure involving PRF, the individual was followed up with after three and six months. Radiographs from future visits revealed that the pocket depth had significantly decreased and the attachment level had increased. According to the author, PRF is a useful regeneration material, particularly for endo-perio lesions. [18] PRF has drawbacks as well. Because it is autologous in nature, the total amount is quite small. [19] The duration of blood sample collection and its transfer to the centrifuge affect PRF's success. For clot polymerization, a glass-coated tube is necessary. It contains the potential for dehydration to decrease PRF and affect its structural integrity. [20] Following root canal therapy, the periodontal discomfort frequently recovers fast in the absence of localised periodontal reasons. Endo-perio abnormalities involve a range of therapeutic options that will result in the most effective infection eradication. [21] A number of therapeutic modalities have been researched for the treatment of people with endo perio problems. [22,23] Researchers have studied the root excision process, retrograde filling procedure, and open flap cleaning procedure. Modern strategies include tissue regeneration procedures like guided tissue regeneration, bone transplants, and the use of tissue growth stimulants (GTR). In this study RAM (relative attachment level) was measured in study participants at 3 months follow up and 6 months follow up. In category one mean change at 3 months was 2.03mm accounting for 32.31% change in RAM. On the other hand mean change at 6 months was 3.63mm accounting for 41.79% change in RAM. When there was evaluation in study participants in category two then it was observed that mean change at 3 months was 2.92 mm accounting for 32.71% change in RAM. On the other hand mean change at 6 months was 3.52 mm accounting for 43.79% change in RAM. There was increase in attachment level periodontal at both 3 months follow up and 6 months follow in both categories; however there was no statistical significant variation observed between the two categories regarding increase in the attachment level on analysis of intergroup variations. [5] Similar histological findings were found in both PRF and T-PRF, according to Tunali et al., however the fibrin clot in T-PRF was denser. 5 T-PRF is superior to PRF in that it persists for increased duration in tissue, forms a denser fibrin matrix, and is more biocompatible thanks to the absence of silica particles. 5 According to research by Tunali et al., research by Chatterjee et al., and research by Mitra et al., there was no statistically substantial variation between the groups as a result of similar histological results. [5,6,7, 8,9,10,11,12,13, 14,15,16,17] T-PRF, however, exhibits more encouraging outcomes. It has been demonstrated that the administration of PRF reduces edoema and discomfort symptoms. [521] Endoperio lesions are a difficult condition to manage, although PRF and fibrin-PRF can be used well. In a study by Monga et al. [22], they found that group which used PRF in the defect had significantly quicker healing after 9 months. Additionally, there is proof that PRF promotes efficient radiographic and clinical bone repair. [23,24,25] The study's drawback is its limited sample size. We only compared PRF and T-PRF. The use of several regenerative substances could have produced beneficial and various outcomes.

## Conclusion:

There was an increase in periodontal attachment and decrease in depth of periodontal pocket in both PRF and T-PRF. However there was no statistical substantial variation observed between the two categories regarding increase in the attachment level or decrease in depth of periodontal when intergroup variations between PRF and T-PRF were considered.

## Figures and Tables

**Table 1:** Probing pocket depth (mm) in both groups

		3 months	6 months
Group I	Mean change	3.21	3.61
	% change	33.79	43.6
Group II	Mean change	2.02	3.62
	% change	34.52	44.01
	t test	0.173	0.236
	p value	0.813	0.703

**Table 2:** Relative attachment level (mm) in both groups

		3 months	6 months
Group I	Mean change	2.03	3.63
	% change	32.31	41.93
Group II	Mean change	2.92	3.52
	% change	32.71	43.23
	t test	0.285	0.289
	p value	0.982	0.989
